# MR findings of microvascular perfusion in infarcted and remote myocardium early after successful primary PCI

**DOI:** 10.1371/journal.pone.0206723

**Published:** 2018-11-09

**Authors:** Anne Bethke, Limalanathan Shanmuganathan, Christian Shetelig, David Swanson, Geir Øystein Andersen, Jan Eritsland, Nils Einar Kløw, Pavel Hoffmann

**Affiliations:** 1 Department of Radiology and Nuclear Medicine, Division of Diagnostics and Intervention, Oslo University Hospital, Ullevål, Oslo, Norway; 2 Institute for Clinical Medicine, Faculty of Medicine, University of Oslo, Oslo, Norway; 3 Feiring Heart Clinic, Feiring, Norway; 4 Department of Cardiology, Oslo University Hospital, Ullevål, Oslo, Norway; 5 Center for Heart Failure Research, Oslo, Norway; 6 Center for Clinical Heart Research, Oslo University Hospital, Oslo, Norway; 7 Institute of Basic Medical Sciences, Department of Biostatistics, University of Oslo, Oslo, Norway; 8 Section for Interventional Cardiology, Department of Cardiology, Oslo University Hospital, Ullevål, Oslo, Norway; Campus Bio-Medico University of Rome, ITALY

## Abstract

**Objectives:**

The aim of the study was to evaluate CMR myocardial first-pass perfusion in the injured region as well as the non-infarcted area in ST-elevation myocardial infarction (STEMI) patients few days after successful primary percutaneous coronary intervention (PCI).

**Materials and methods:**

220 patients with first time STEMI successfully treated with PCI (with or without postconditioning) were recruited from the Postconditioning in STEMI study. Contrast enhanced CMR was performed at a 1.5 T scanner 2 (1–5) days after PCI. On myocardial first-pass perfusion imaging signal intensity (SI) was measured in the injured area and in the remote myocardium and maximum contrast enhancement index (MCE) was calculated. MCE = (peak SI after contrast—SI at baseline) / SI at baseline x 100.

**Results:**

There were no significant differences in first-pass perfusion between patients treated with standard PCI and patients treated with additional postconditioning. The injured myocardium showed a significantly lower MCE compared to remote myocardium (94 ± 55 vs. 113 ± 49; p < 0.001). When patients were divided into four quartiles of MCE in the injured myocardium (MCE injured myocardium), patients with low MCE injured myocardium had: significantly lower ejection fraction (EF) than patients with high MCE injured myocardium, larger infarct size and area at risk, smaller myocardial salvage and more frequent occurrence of microvascular obstruction on late gadolinium enhancement. MCE in the remote myocardium revealed that patients with larger infarction also had significantly decreased MCE in the non-infarcted, remote area.

**Conclusion:**

CMR first-pass perfusion can be impaired in both injured and remote myocardium in STEMI patients treated with primary PCI. These findings indicate that CMR first-pass perfusion may be a feasible method to evaluate myocardial injury after STEMI in addition to conventional CMR parameters.

## Introduction

In patients with acute ST-elevation myocardial infarction (STEMI) the recommended treatment to restore coronary blood flow is percutaneous coronary intervention (PCI) within the first 120 minutes after the STEMI diagnosis has been made [1]. After successful PCI, normal TIMI (Thrombolysis in Myocardial Infarction) flow in the infarct related artery (IRA) post procedure does not always translate into sufficient microcirculation at the myocardial level [2, 3]. Thus, visualization of the patency of the IRA alone is not sufficient for scheduling treatment and physiological aspects are essential. Previous studies have shown that TIMI myocardial perfusion (TMP) may provide additional prognostic information to TIMI flow grade [2, 4, 5].

Cardiac magnetic resonance (CMR) imaging is a sensitive method for evaluation of the microcirculation with high spatial resolution [6, 7]. Over the last two decades CMR imaging has been used in STEMI patients to evaluate treatment and to predict and measure final outcome after the infarction [8–10]. Common CMR parameters reported are left-ventricular end-diastolic volume (EDV), end-systolic volume (ESV) and ejection fraction (EF), infarct size (IS) by late gadolinium enhancement (LGE), microvascular obstruction (MVO), area at risk (AaR) and myocardial salvage [11]. LGE is regarded as the gold standard to predict left ventricular remodeling [12–14] and MVO is associated with the outcome in STEMI patients [15]. First-pass perfusion has been shown to be more sensitive to detect alteration of the microvascular perfusion than early and late gadolinium enhancement [16, 17]. First-pass perfusion is therefore suitable as a supplementary method to LGE and MVO as it might also detect less pronounced microvascular damage missed by other methods [18].

This study included patients from the Postconditioning in STEMI (POSTEMI) study. Compared with conventional primary PCI, PCI combined with ischemic postconditioning did not influence the primary endpoint of infarct size nor the secondary endpoints of MVO, myocardial salvage and ejection fraction, as determined by CMR [19]. The aim of the present study was to evaluate CMR myocardial first-pass perfusion in the injured as well as the non-infarcted area in STEMI patients few days after successful primary PCI.

## Material and methods

### Study population and treatment protocol

The study population was recruited from the POSTEMI study (ClinicalTrials.gov Po1506) on patients with acute STEMI treated with PCI with or without a postconditioning procedure [20]. The study protocol was approved by the Regional Committee for Medical Research Ethics, South-East Norway and all patients gave written informed consent to participate in the study.

The study protocol is described in detail elsewhere [19, 20]. In brief, patients with acute STEMI with symptoms of less than 6 hours duration and occlusion of a proximal or mid-coronary artery with TIMI flow 0 or 1 before PCI were included in the POSTEMI study. Main exclusion criteria were previous myocardial infarction, occlusion of other coronary arteries than the IRA, ongoing treatment for angina pectoris, thrombolysis given as primary reperfusion treatment, cardiogenic shock, pulmonary congestion, severe hypotension, renal failure (serum creatinine >200 mmol/L) and general contraindication to MRI.

Patients were treated with primary PCI (standard treatment) or primary PCI with postconditioning. The postconditioning was performed with balloon inflations, 4 cycles of 1 min reocclusion and 1 min reperfusion, starting 1 min after opening of the occluded coronary artery [21].

All patients were treated with a stent in the IRA and received a GPIIb/IIIa inhibitor.

**Fig 1:**
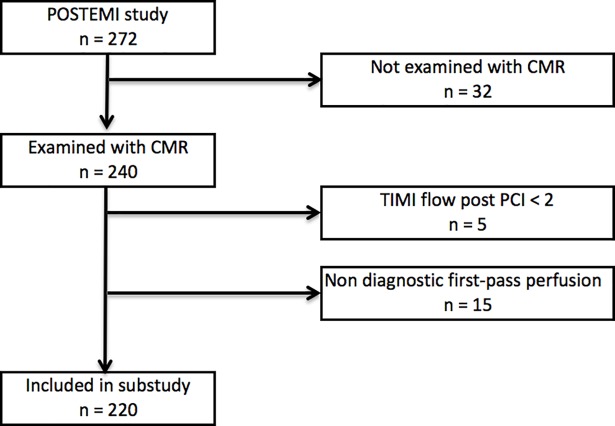
Study flow diagram. CMR (cardiac MRI), PCI (percutaneous coronary intervention), TIMI (Thrombolysis in Myocardial Infarction).

Of the 272 patients randomized in the POSTEMI trial, 220 patients were included in the present study (Fig 1). Reasons for not performing an early CMR examination (n = 32) were: not scanner capacity before patient discharge (n = 14); claustrophobia (n = 4); reduced clinical presentation (n = 3); reduced compliance (n = 3); death (n = 2); MRI contraindications (n = 2), high body weight (n = 1) or unknown (n = 3). Of the remaining 240 patients five patients had TIMI flow 0–1 post PCI or non diagnostic first-pass perfusion imaging (n = 15) and were excluded. 220 patients with a CMR examination of diagnostic quality were included in this substudy.

### Clinical follow-up data

Clinical data were collected at one year and a composite endpoint was defined as death, myocardial infarction, unscheduled revascularization >3 months after the index infarction, rehospitalization for heart failure, or stroke.

### CMR protocol

CMR was performed within 2 (1–5) days after the STEMI on a 1.5 T scanner (Philips Intera, release 11 or Philips Achieva, release 3.2.1.1; Philips Healthcare, Best, The Netherlands), using five-element synergy-cardiac coil or Sense-cardiac coil, respectively, and vector-based electrocardiography (ECG).

For cine imaging the left ventricle was scanned in two-, three- and four-chamber long axis view using balanced fast-field echo sequences for functional analysis, and short axis images were acquired for complete left ventricular volume analysis. T2-weighted imaging was performed with five short axis slices, covering the ventricle from basis to apex using black blood inversion recovery fast spin echo sequences. The most apical and the most basal slices were rejected and the three mid slices were used for analyses.

First-pass perfusion at rest was performed using ECG triggered fast T1 enhanced gradient-echo technique with segmented K-space and saturation pre-pulse. Three short axis slices were acquired during breath hold, scanning for one minute. The parameters were: TR (time to repeat) 3.2 ms, TE (time to echo) 1.26 ms, flip angle 20° and saturation prepulse delay 200 ms, trigger delay approximately 210 ms, matrix 128 and field of view 350 mm, slice thickness 10 mm, gap 20 mm.

The LGE study was performed 15 minutes after the first-pass study in short axis and two- and four-chamber long axis view, using 3D turbo-field-echo technique with inversion pre-pulses, covering the whole left ventricle. The imaging parameters were: TR 4.5 ms, TE 1.44 ms, flip angle 15° and trigger delay 375–1226 ms, chosen according to the ECG RR interval to fit end-diastole. The optimal T1 for suppression of the myocardium was achieved by the Look-Locker sequence before the diagnostic sequences was performed [22].

In all patients, gadolinium-DTPA (Magnevist, Schering AG, Germany) was injected using Spectris power injector. For the first-pass perfusion imaging, 0.05 mmol/kg body weight was injected at the rate of 3 ml/s into the left brachial vein, followed by 25 ml of saline infused at the same speed [23]. For LGE imaging, an additional dose of 0.1 mmol/kg of Magnevist was injected 15 minutes before acquisition.

### CMR analysis

All image analysis was performed offline on a View Forum workstation, Philips Intera extended MR workspace R2.6.3.1. (Philips Medical Systems, The Netherlands). First-pass perfusion imaging was analyzed by one reader (AB) with 5 years experience. All other parameters were analyzed by another reader (PH) with an experience >10 years within the field.

EF was calculated by assessment of the volumes of the endocardial contours in diastole and systole of the short axis images as per standard practice [11].

AaR was assessed in the T2 images as myocardium with a SI of more than 2 standard deviations above the signal intensity (SI) in remote myocardium. The contour of the area with hyperintense signal was than manually drawn in the central 3 of the 5 short axis slices. As the remote area, myocardium supplied by other arteries than the IRA remote to the infarction was chosen. High-grade stenosis in the remote area was ruled out via coronary angiography findings. Myocardial hemorrhage was assessed visually as a hypoenhanced region within the AaR on T2 imagages. The size of the hemorrhage was not assessed.

**Fig 2:**
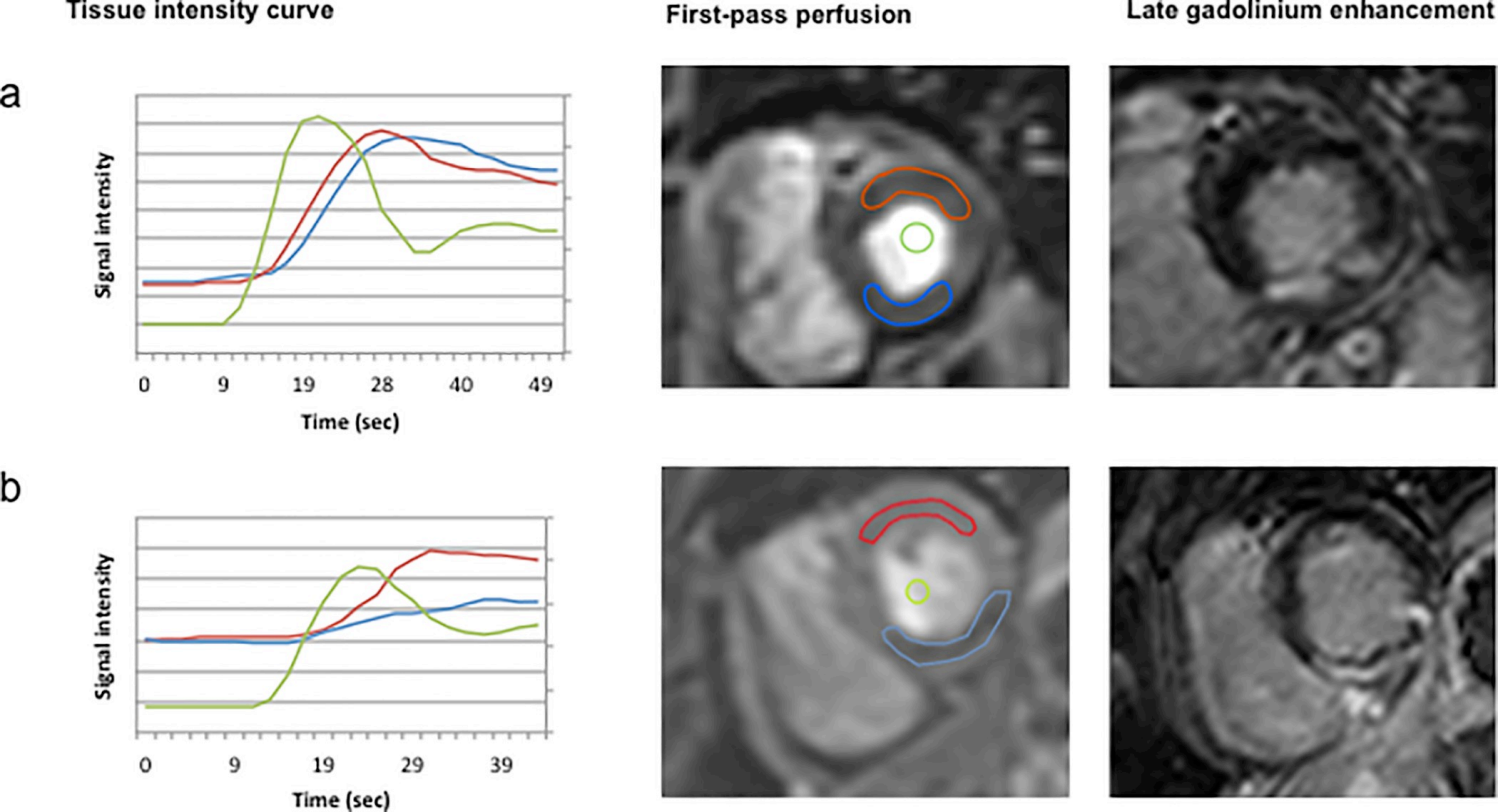
Analysis of first-pass perfusion CMR. Fig 2A shows perfusion analysis of a patient with an inferior wall infarction with high MCE in the injured and remote myocardium. Fig 2B shows decreased perfusion in the injured myocardium in the inferior wall compared to the remote area. On the LGE study a hypointens area in the endocardium indicates MVO. Green line: ROI blood pool in the left ventricle, blue line: ROI injured myocardium, red line: ROI remote myocardium. Because of the different magnitude of the signal in the blood pool and myocardium a secondary vertical axis has been implemented.

Myocardial first-pass perfusion was analyzed semi-quantitatively by measuring the SI versus time [11]. SI was measured with manually traced regions of interest in the lumen of the left ventricle, the area of the injured myocardium and in a myocardial area remote to the infarction [6] (Fig 2). The region of the injured myocardium was specified by visual comparison with the LGE study, also including the MVO area. The short axis slice with the greatest infarct extension was considered being the most representative of the infarction. For the lumen of the left ventricle maximum SI was given. The following parameters were calculated for injured and remote myocardium: $Maximum\;contrast\;enhancement\;index\;(MCE)=\frac{\left(peak\;SI\;after\;contrast–SI\;at\;baseline\right)}{SI\;at\;baseline}\times 100$ [24–26]. Time to peak contrast enhancement (TTP) was visually assessed and measured from entry of the contrast medium into the left ventricle to peak myocardial SI [27]. Additionally, $\frac{MCE}{TTP}$ was calculated for each region.

The IS was assessed as a volume in the LGE images by manually drawing the contour of the hyperenhanced area in short axis slices [11]. The volume of the infarction was also related to the total left ventricular myocardial volume and given in percentage. Presence of MVO was defined as a hypointense area in the center of the hyperintense area representing infarcted myocardium, assessed in LGE images. The size of MVO was not measured. Myocardial salvage was defined as $\frac{\left(AaR–IS\right)}{AaR}\times 100$ [28].

The readers were blinded to treatment strategy and clinical outcome. The intraobserver reliability estimated by the intraclass correlation coefficients for myocardium at risk and infarct size were 0.876, 95% CI (0.628 to 0.963) and 0.985, 95% CI (0.963 to 0.994), respectively [19].

Within observer consistency and agreement was 0.902, 95% CI (0.716–0.972) and 0.906, 95% CI (0.705–0.972) for MCE injured myocardium and 0.878, 95% CI (0.782–0.962) and 0.883, 95% CI (0.774–0.963) for MCE remote myocardium. The Pearson`s correlation coefficient between observers was 0.960, 95% CI (0.903–0.984) for MCE injured myocardium and 0.759, 95% CI (0.488–0.897).

Consistency and agreement between observers was: 0.958, 95% CI (0.919–0.981) and 0.960, 95% CI (0.915–0,980) for MCE injured myocardium and 0.741, 95% CI (0.395–0.981) and 0.741, 95% CI (0.381–0.981) for MCE remote myocardium, respectively.

### Design and statistical analysis

This substudy was a cohort study of exposed (standard treatment and postconditioning) versus unexposed (standard treatment) patients.

Peak troponin is given as median (interquartile range) as the distribution was right skewed. Other continuous data were approximately normally distributed and are presented as mean ± standard deviation, binary data as distribution of categories.

To compare patients with different perfusion levels, MCE data of injured myocardium (MCE injured myocardium) were divided into 4 quartiles where quartile 1 represents the highest and quartile 4 the lowest perfusion level. The one-way ANOVA or the chi-square test for categorical variables were used to compare the means of the groups.

Normality and homoscedasticity of the residuals was confirmed for MCE injured myocardium (S1 Fig). Therefore, linear regression analysis was performed for continuous outcomes (dependent variable: EF, ESV, EDV, IS, AaR, myocardial salvage) and logistic regression for binary outcomes (dependent variables: MVO, myocardial hemorrhage). We additionally adjusted for confounding by including age, sex, symptom-to-balloon time, IRA, smoking status, diabetes, treatment for dyslipidemia into all our models. Examination of residual plots of MCE show approximately normally distributed and homoskedastic errors as well as linearity in the explanatory variable, making further variable transformation unnecessary.

Correlation between MCE injured myocardium and the composite endpoint was done with Spearman´s rho.

A p-value <0.05 was considered statistically significant. We used IBM SPSS Statistics 24 for all analysis.

## Results

Baseline characteristics of the 220 patients included in this substudy are shown in Table 1. Except for patient age (standard treatment 58.4 ± 10.1; postconditioning 61.6 ±11, p = 0.026) there were no significant differences in baseline parameters, or in CMR parameters, including first-pass perfusion, between the two treatment groups (MCE injured myocardium: standard treatment 92 ± 84; postconditioning 95 ± 74, p = 0.659). Therefore, groups were merged for further analysis.

**Table 1:** Patient baseline characteristics.

** n (total)**		220
**Age (years)**		60 ± 11
**Female gender**		37 (17)
**Peak troponin T, ng/l**		5643 (2311 to 8974)
**Infarct related artery**	**LAD**	108 (49)
	**LCX**	23 (10)
	**RCA**	89 (40)
**1-vessel disease**		140 (64)
**2-vessel disease**		55 (25)
**3-vessel disease**		25 (11)
**Current and former smoking**		146 (66)
**Treated hypertension**		60 (27)
**Diabetes**		13 (5.9)
**Dyslipidemia**		23 (10)

Peak troponin is presented in median (interquartile range); age is presented as mean ± standard deviation, other data as n (%). LAD (left anterior descending artery), LCX (left circumflex artery), RCA (right coronary artery)

CMR first-pass perfusion revealed significantly reduced MCE and delayed TTP in the injured myocardium compared to remote myocardium (MCE: 94 ± 55 vs. 113 ± 49, p< 0.001; TTP 15.8 ± 4.9 sec vs. 14.8 ± 4.1 sec, p< 0.001).

When MCE data of the injured myocardium were divided into four quartiles, peak troponin T was higher for patients with lower MCE. However, there were no significant differences in the angiographic data like symptom-to-balloon time, door-to-balloon time, IRA, multi vessel disease, TIMI flow post PCI or the use of thrombectomy related to the level of MCE (Table 2).

**Table 2:** Clinical, biochemical and coronary angiographic data according to quartiles of MCE in the injured myocardium. Quartile 1 represents the highest MCE and quartile 4 the lowest MCE level.

Quartiles of MCE in the injured myocardium		1	2	3	4	p value
**Peak Troponin T, ng/l**		4446 (2075 to 6817)	5297 (2286 to 8308)	6508 (2913 to 10103)	7760 (3913 to 11608)	<0.001
**Symptom-to-balloon time, minutes**		186 ± 89	200 ± 95	208 ± 89	193 ± 75	0.590
**Door-to-balloon time, minutes**		31 ± 8.3	35 ± 15	36 ± 11	35 ± 11	0.169
**Infarct related artery **	**LAD**	26 (47)	32 (58)	23 (42)	27 (49)	0.331
	**LCX**	4 (7.3)	3 (5.5)	7 (13)	9 (16)	
	**RCA**	25 (46)	20 (36)	25 (46)	19 (35)	
**1-vessel disease**		31 (56)	38 (69)	35 (64)	36 (66)	0.832
**2-vessel disease**		18 (33)	11 (20)	13 (24)	13 (24)	
**3-vessel disease**		6 (11)	6 (11)	7 (13)	6 (11)	
**TIMI flow post PCI**	**2**	2 (3.6)	3 (5.5)	4 (7.3)	1 (1.8)	0.553
	**3**	53 (96)	52 (95)	51 (93)	54 (98)	
**Thrombectomy**		13 (24)	11 (20)	12 (22)	12 (22)	0.975

Peak troponin is presented in median (interquartile range). Other data are presented mean ± standard deviation or as n (% within the MCE groups). MCE (maximum contrast enhancement index), LAD (left anterior descending artery), LCX (left circumflex artery), RCA (right coronary artery), TIMI (Thrombolysis in Myocardial Infarction). p-values indicate level of significance between the 4 quartiles

Comparing the level of MCE to the functional CMR parameters, patients with high MCE injured myocardium (1. quartile) had significantly smaller EDV and ESV and higher EF compared to patients with low MCE injured myocardium (4. quartile) (Table 3). Comparing the level of MCE injured myocardium to the other CMR parameters of infarction, patients with high MCE injured myocardium had smaller IS, smaller AaR and larger myocardial salvage. In patients with high MCE injured myocardium, myocardial hemorrhage and MVO on LGE was significantly less frequent than in patients with low MCE injured myocardium. The differences were significant also when controlled for confounders (age, sex, symptom-to-balloon time, IRA, smoking status, treated dyslipidemia) (Table 4).

**Table 3:** CMR data according to quartiles of MCE of the injured myocardium.

**Quartiles of MCE injured myocardium**	1	2	3	4	
n = 220	55	55	55	55	p-value
**MCE injured myocardium**	**171 ± 38**	**101 ± 12**	**67 ± 7.5**	**36 ± 13**	
MCE remote myocardium	161 ± 47	119 ± 36	97 ± 36	76 ± 31	< 0.001
TTP injured myocardium	15 ± 3.8	16 ±5.4	16 ± 5.9	16 ± 4.4	0.545
TTP remote myocardium	12 ± 3.4	15 ± 5	15 ± 3.8	16 ± 3.9	0.323
MCE/TTP injured myocardium	12 ±4.1	7 ±2	4.5 ±1.2	2.3 ± 1	< 0.001
MCE/TTP remote myocardium	12 ± 4.4	8.8 ± 3.7	7.3 ±3.7	5.3 ± 2.9	< 0.001
EDV	152 ± 37	172 ± 46	178 ± 47	182 ± 36	0.001
ESV	69 ± 27	89 ± 46	90 ± 39	95 ± 31	0.001
EF	55 ± 8.9	50 ± 13	51 ± 11	48 ± 11	0.009
Total infarct volume	18 ± 14	27 ± 20	31 ± 24	37 ± 25	< 0.001
Relative infarct volume, (%)	14 ± 9.5	20 ± 14	21 ± 13	24 ± 14	0.001
Area at risk, (%)	37 ± 13	44 ± 16	45 ± 15	44 ±12	0.023
Myocardial salvage	39 ± 29	37 ± 19	28 ± 18	28 ± 22	0.016
MVO on LGE, n (%)	12 (23)	23 (43)	27 (49)	40 (74)	< 0.001
Myocardial hemorrhage, n (%)	8 (16)	15 (29)	26 (49)	30 (57)	< 0.001
Days between PCI and CMR	2.1 ± 1.2	2.1 ± 0.9	2.1 ± 1.1	2.2 ± 1	0.963

Data are presented as mean ± standard deviation, except MVO and hemorrhage, n (%). TTP (time to peak), MCE (maximum contrast enhancement index), EDV (end-diastolic volume), ESV (end-systolic volume), EF (ejection fraction), MVO on LGE (microvascular obstruction on late gadolinium enhancement images). p-values indicate level of significance between the 4 quartiles

**Table 4:** Regression analysis to control for confounding effects on the association between MCE injured myocardium and other CMR outcome variables (dependent variable).

Dependent variable	B	p-value
EDV	-0.133	0.007
ESV	-0.11	0.009
EF	0.027	0.032
Relative infarct volume	-0.047	0.001
Area at risk	-0.041	0.014
Myocardial salvage	0.068	0.025
MVO on LGE	-0.015	<0.001
Myocardial hemorrhage	-0.015	<0.001

Independent variable: MCE injured myocardium. Variables controlled for were: age, sex, symptom-to-balloon time, infarct related artery, smoking status, diabetes and treatment dyslipidemia. EDV (end-diastolic volume), ESV (end-systolic volume), EF (ejection fraction), MVO on LGE (microvascular obstruction on late gadolinium enhancement images)

Peak SI in the lumen of the left ventricle was not significantly different between the four groups (quartile 1: 2823; quartile 2: 2535; quartile 3: 2524, quartile 4: 2359; p = 0.102).

Analyzes of MCE in the remote myocardium showed that in the 1. quartile MCE in the remote myocardium tended to be lower (not significant) than in the injured myocardium. In all the other quartiles of MCE injured myocardium, MCE remote myocardium was higher than MCE injured myocardium. Data also showed that MCE remote myocardium decreased significantly from the 1. to the 4. quartile (161 ± 47 vs. 76 ± 31, p = < 0.001).

Clinical events were few. Of the 220 patients included in the present substudy 13 patients had a clinical event as defined in the composite endpoint. There was no significant difference between the four groups of MCE injured myocardium (Table 5).

**Table 5:** Composite endpoints according to quartiles of MCE of the injured myocardium.

Quartiles of MCE injured myocardium	1	2	3	4	Spearman correlation coefficient	p-value
Composite endpoint	3 (5,5)	6 (10,9)	2 (3,6)	2 (3,6)	-0,06	0,373

Data are presented as n (%). Composite endpoints were composed of death, myocardial infarction, unscheduled revascularization >3 months after the index infarction, rehospitalization for heart failure, or stroke.

## Discussion

In the present study, the results on CMR first-pass perfusion at rest showed no significant differences between patients treated with PCI with or without postconditioning, which is in accordance with the findings in the main study [19, 29].

Normalized flow in the epicardial arteries, in the absence of high-grade stenosis, gives a comparable blood supply to the entire myocardium. Still, microvascular perfusion in the injured myocardium was significantly lower than in the remote area of the left ventricle. The main findings of the present study are, that the level of MCE injured myocardium was associated with EDV, ESV and EF as well as the IS, AaR, myocardial salvage and the occurrence of MVO at LGE imaging. Furthermore, in patients with low perfusion in the injured myocardium also perfusion in the remote myocardium was significantly impaired. Semiquantitative parameters do not depend on the relative difference between injured and remote myocardium and therefore minor changes in remote myocardium can be detected. Recently, research on STEMI patients has focused more on the remote myocardium [30] and parameters evaluating the remote myocardium in an early state after STEMI may be of value when new treatments are tested.

In patients with acute STEMI and restored TIMI 3 flow in the IRA, TMP grade assessed at final angiography has been shown to have prognostic value [2, 4]. The present study focuses on the association between microvascular perfusion assessed with CMR first-pass perfusion and the early outcome in STEMI patients. MVO on CMR is widely regarded as a manifestation of reperfusion injury after STEMI [31, 32]. However, MVO is reported in over 50% of patients post STEMI reperfused by PCI irrespective of post procedural TIMI flow grade [3, 10]. CMR first-pass perfusion is a suitable tool to evaluate microperfusion, as it is more sensitive than MVO on LGE regarding the blood supply to the microvasculature [16, 18].

Despite standardized contrast media dose and infusion rate as well as examination technique and TIMI 3 flow after PCI in the majority of the patients (95.5%), there were significant differences in CMR first-pass perfusion at rest. In the present study, patients with high MCE injured myocardium performed better on functional CMR parameters and presented with significantly smaller IS, and even though they had smaller AaR, they also had higher myocardial salvage. This is in agreement with the findings of Hopp et al. [7], who also found a correlation between peak enhancement in the infarcted myocardium and the presence of MVO at LGE. Ørn et al. also demonstrated larger infarct sizes at six months in the presence of impaired first-pass perfusion [33]. CMR first-pass perfusion might therefore be a more sensitive parameter for evaluation of new treatment strategies [18, 34]. In a recent study Lombardo and coworkers showed a correlation between the extent and transmurality of MVO on first-pass perfusion imaging and left ventricular remodeling [35].

The present study also shows that MCE remote myocardium may be impaired after STEMI treated with PCI. As none of the other CMR parameters in our study, such as T2, LGE or cine sequences showed alterations in the myocardium supplied of other than the target vessel, first-pass perfusion appears to be a more sensitive parameter for evaluating the microvasculature. Also previous CMR studies have found alterations in perfusion of the myocardium adjacent to the infarction and also in the remote myocardium. For instance, Bodi et al. found hypoperfusion in the non-infarcted myocardium in 13% of the myocardial segments adjacent to the infarction [36] and Taylor et al. describes longer TTP in myocardial areas surrounding the infarcted area [37]. Further, Nagao et al. found reduced myocardial perfusion in the remote myocardium in patients with anterior wall infarction [38]. Rogers et al. [39] showed a non-significant improvement of perfusion in the non-infarcted myocardium after myocardial infarction from week one to week seven by 24% in a small study of 17 patients. Stunning might be considered as a possible explanation for these findings [9]. Based on a PET/MRI animal study Lee and colleagues assume a correlation of inflammation in the remote myocardium post STEMI and left ventricular remodeling [40]. Another potential cause could be earlier unnoticed infarction in other vascular territories, although previous infarction and occlusion of a non-infarct related artery was an exclusion criterion in our study. Furthermore, there was no evidence of other infarction on LGE. Non-culprit lesion or collateral flow reduction might cause deprived first-pass perfusion in the remote myocardium [16, 41]. However, coronary stenosis do normally not reduce resting myocardial perfusion [42]. The differences in MCE injured myocardium and MCE remote myocardium were not caused by a more complex coronary disease in patients with lower MCE injured myocardium as the percentage of two- and three-vessel disease was similar between the groups and also TIMI flow post PCI was not significantly different between the groups. As indicated by similar maximum SI in the blood pool in the left ventricle, the differences in MCE between groups were not due to infusion variations or because of individual hemodynamics.

Evolving techniques like T1 mapping have also shown changes in the remote myocardium [30, 43, 44]. Carberry and colleagues demonstrated an association between the extracellular volumes in the remote zone and left ventricular EF [30]. As the changes in ECV in the remote zone were consistent over six months and not accompanied by T2 changes they concluded that underlying replacement fibrosis is a probable explanation. Further, Reinstadler and colleagues found a correlation of native T1 values in the remote zone and major adverse cardiac events [43].

The remote myocardium in patients with STEMI has also been evaluated in a series of studies applying other methods than CMR. In a recent study in patients with acute myocardial infarction, Cheng et al. [45] showed a correlation between impaired coronary flow reserve measured by contrast enhanced echocardiography in the non-infarcted myocardium and left ventricular remodeling and function. Decreased microvascular density and activation of the renin-angiotensin system were proposed as underlying mechanism [45, 46]. Another theory implies that in the situation of an acute infarction the remote myocardium initiates compensatory metabolic reactions [47]. Other studies reveal reduced coronary flow reserve in the infarcted as well as in the remote myocardium in the acute phase of AMI, both with intracoronary measurements [48] and by [15O]H2O positron emission tomography [49]. Impaired vasodilation capacity [47] and dysfunctional autoregulation of the microvasculature caused by neurohumoral activation might in part explain these findings [48].

Echocardiography with circumferential strain could as well show a significant correlation between alterations in the non-infarcted myocardium and diastolic function [50]. It was assumed that compensatory hypercontractility in the remote myocardium in case of STEMI contributes essentially to these findings.

Several histological studies, both in animals and patients found alterations like focal necrosis not only in the infarcted but also in the remote myocardium early after myocardial infarction [51, 52]. Olivetti et al. for instance found apoptosis in 12% of the cardiomyocytes in the AaR and in about 1% in the remote myocardium in a study on patients with large infarctions [53]. These changes in the remote myocardium might contribute to the alterations seen in the present study of CMR first-pass perfusion. However, the data of the present study does not imply a clear explanation and the findings in the remote myocardium remain an observation.

STEMI patients treated with primary PCI do generally have a good prognosis [54, 55]. In our study only 5,9% of the patients had an adverse clinical event within the first year and no significant difference between the MCE groups was found. However, the study was not powered to detect differences in clinical events and one has to be cautious to draw conclusions. As clinical events are rare, reliable surrogate endpoints are needed in studies evaluating treatment regimes in STEMI patients. MCE of the injured and remote myocardium may be used in future trials as a surrogate to reduce sample size.

However, to prevent left ventricular remodeling patients at high risk and in need for intensified therapy need to be identified at an early point in time. The results of the present study indicate that changes in the remote and injured myocardium are related to outcome. As LGE normally does not show alteration in the remote myocardium, we additionally to the standard parameters propose MCE in the injured and remote myocardium as an early marker for risk stratification in STEMI patients treated with primary PCI. However, more standardization of perfusion in the remote myocardium is needed before clinical application. This can be an interesting research area for future investigations.

### Limitations

In the present study first-pass perfusion CMR covers only 3 slices and not the entire left ventricle, which can lead to inaccuracy [16]. Great care was taken to standardize contrast infusion, but we cannot completely exclude some variations in infusion and hemodynamics between individual patients.

SI can vary within different distances to the coil. We accounted for that by relating peak SI to baseline SI. Moreover, IRA was evenly distributed between the different groups.

We applied a semiquantitative analysis for the evaluation of first-pass perfusion imaging instead of analyzing the absolute values. However, semiquantitative approaches have been shown to be valuable in STEMI patients [7, 37].

## Conclusion

CMR first-pass perfusion can be impaired in both injured and remote myocardium in STEMI patients treated with primary PCI. Impaired first-pass perfusion in the remote myocardium was particularly seen when impairment was larger in the injured myocardium. First-pass perfusion provided additional information to TIMI flow at angiography.

## Supporting information

S1 Fig Residuals plot MCE injured myocardium. (TIF)

S1 Table (XLSX)

## References

[1] Ibanez B, James S, Agewall S, Antunes MJ, Bucciarelli-Ducci C, Bueno H, et al 2017 ESC Guidelines for the management of acute myocardial infarction in patients presenting with ST-segment elevation: The Task Force for the management of acute myocardial infarction in patients presenting with ST-segment elevation of the European Society of Cardiology (ESC). Eur Heart J. 2017 10.1093/eurheartj/ehx393 . 28886621

[2] Appelbaum E, Kirtane AJ, Clark A, Pride YB, Gelfand EV, Harrigan CJ, et al Association of TIMI myocardial perfusion grade and ST-segment resolution with cardiovascular magnetic resonance measures of microvascular obstruction and infarct size following ST-segment elevation myocardial infarction. J Thromb Thrombolysis. 2009;27(2):123–9. 10.1007/s11239-008-0197-y . 18246410

[3] Nijveldt R, Beek AM, Hirsch A, Stoel MG, Hofman MB, Umans VA, et al Functional recovery after acute myocardial infarction: comparison between angiography, electrocardiography, and cardiovascular magnetic resonance measures of microvascular injury. J Am Coll Cardiol. 2008;52(3):181–9. 10.1016/j.jacc.2008.04.006 . 18617066

[4] Bethke A, Halvorsen S, Bohmer E, Abdelnoor M, Arnesen H, Hoffmann P. Myocardial perfusion grade predicts final infarct size and left ventricular function in patients with ST-elevation myocardial infarction treated with a pharmaco-invasive strategy (thrombolysis and early angioplasty). 2015;11(5):518–24. 10.4244/EIJY15M04_02 . 25868877

[5] Gibson CM, Cannon CP, Murphy SA, Marble SJ, Barron HV, Braunwald E, et al Relationship of the TIMI myocardial perfusion grades, flow grades, frame count, and percutaneous coronary intervention to long-term outcomes after thrombolytic administration in acute myocardial infarction. Circulation. 2002;105(16):1909–13. . 11997276 10.1161/01.cir.0000014683.52177.b5

[6] Hoffmann P, Halvorsen S, Stensaeth KH, Brekke M, Muller C, Anker GO, et al Myocardial perfusion in ST-elevation myocardial infarction treated successfully with primary angioplasty. Scand Cardiovasc J. 2006;40(2):96–104. 10.1080/14017430600628144 . 16608779

[7] Hopp E, Bjornerud A, Lunde K, Solheim S, Aakhus S, Arnesen H, et al Perfusion MRI at rest in subacute and chronic myocardial infarct. Acta radiologica. 2013;54(4):401–11. 10.1177/0284185113475605 . 23401603

[8] Wu KC, Zerhouni EA, Judd RM, Lugo-Olivieri CH, Barouch LA, Schulman SP, et al Prognostic significance of microvascular obstruction by magnetic resonance imaging in patients with acute myocardial infarction. Circulation. 1998;97(8):765–72. . 9498540 10.1161/01.cir.97.8.765

[9] Wu E, Ortiz JT, Tejedor P, Lee DC, Bucciarelli-Ducci C, Kansal P, et al Infarct size by contrast enhanced cardiac magnetic resonance is a stronger predictor of outcomes than left ventricular ejection fraction or end-systolic volume index: prospective cohort study. Heart. 2008;94(6):730–6. 10.1136/hrt.2007.122622 . 18070953

[10] van Kranenburg M, Magro M, Thiele H, de Waha S, Eitel I, Cochet A, et al Prognostic value of microvascular obstruction and infarct size, as measured by CMR in STEMI patients. JACC Cardiovasc Imaging. 2014;7(9):930–9. 10.1016/j.jcmg.2014.05.010 . 25212799

[11] Schulz-Menger J, Bluemke DA, Bremerich J, Flamm SD, Fogel MA, Friedrich MG, et al Standardized image interpretation and post processing in cardiovascular magnetic resonance: Society for Cardiovascular Magnetic Resonance (SCMR) board of trustees task force on standardized post processing. J Cardiovasc Magn Reson. 2013;15:35 10.1186/1532-429X-15-35 ; PubMed Central PMCID: PMCPMC3695769. 23634753 PMC3695769

[12] Nijveldt R, Hofman MB, Hirsch A, Beek AM, Umans VA, Algra PR, et al Assessment of microvascular obstruction and prediction of short-term remodeling after acute myocardial infarction: cardiac MR imaging study. Radiology. 2009;250(2):363–70. 10.1148/radiol.2502080739 . 19164698

[13] Baks T, van Geuns RJ, Biagini E, Wielopolski P, Mollet NR, Cademartiri F, et al Recovery of left ventricular function after primary angioplasty for acute myocardial infarction. Eur Heart J. 2005;26(11):1070–7. 10.1093/eurheartj/ehi131 . 15716283

[14] Larose E, Rodes-Cabau J, Pibarot P, Rinfret S, Proulx G, Nguyen CM, et al Predicting late myocardial recovery and outcomes in the early hours of ST-segment elevation myocardial infarction traditional measures compared with microvascular obstruction, salvaged myocardium, and necrosis characteristics by cardiovascular magnetic resonance. J Am Coll Cardiol. 2010;55(22):2459–69. 10.1016/j.jacc.2010.02.033 . 20510213

[15] Durante A, Laricchia A, Benedetti G, Esposito A, Margonato A, Rimoldi O, et al Identification of High-Risk Patients After ST-Segment-Elevation Myocardial Infarction: Comparison Between Angiographic and Magnetic Resonance Parameters. Circ Cardiovasc Imaging. 2017;10(6):e005841 10.1161/CIRCIMAGING.116.005841 . 28592591

[16] Mather AN, Lockie T, Nagel E, Marber M, Perera D, Redwood S, et al Appearance of microvascular obstruction on high resolution first-pass perfusion, early and late gadolinium enhancement CMR in patients with acute myocardial infarction. J Cardiovasc Magn Reson. 2009;11:33 10.1186/1532-429X-11-33 ; PubMed Central PMCID: PMCPMC2733303. 19698105 PMC2733303

[17] Bogaert J, Kalantzi M, Rademakers FE, Dymarkowski S, Janssens S. Determinants and impact of microvascular obstruction in successfully reperfused ST-segment elevation myocardial infarction. Assessment by magnetic resonance imaging. Eur Radiol. 2007;17(10):2572–80. 10.1007/s00330-007-0627-9 . 17361420

[18] Yan AT, Gibson CM, Larose E, Anavekar NS, Tsang S, Solomon SD, et al Characterization of microvascular dysfunction after acute myocardial infarction by cardiovascular magnetic resonance first-pass perfusion and late gadolinium enhancement imaging. J Cardiovasc Magn Reson. 2006;8(6):831–7. 10.1080/10976640600778049 . 17060106

[19] Limalanathan S, Andersen GO, Klow NE, Abdelnoor M, Hoffmann P, Eritsland J. Effect of ischemic postconditioning on infarct size in patients with ST-elevation myocardial infarction treated by primary PCI results of the POSTEMI (POstconditioning in ST-Elevation Myocardial Infarction) randomized trial. J Am Heart Assoc. 2014;3(2):e000679 10.1161/JAHA.113.000679 ; PubMed Central PMCID: PMC4187468. 24760962 PMC4187468

[20] Limalanathan S, Andersen GO, Hoffmann P, Klow NE, Abdelnoor M, Eritsland J. Rationale and design of the POSTEMI (postconditioning in ST-elevation myocardial infarction) study. Cardiology. 2010;116(2):103–9. 10.1159/000316965 . 20588018

[21] Staat P, Rioufol G, Piot C, Cottin Y, Cung TT, L'Huillier I, et al Postconditioning the human heart. Circulation. 2005;112(14):2143–8. 10.1161/CIRCULATIONAHA.105.558122 . 16186417

[22] Doltra A, Amundsen BH, Gebker R, Fleck E, Kelle S. Emerging concepts for myocardial late gadolinium enhancement MRI. Curr Cardiol Rev. 2013;9(3):185–90. 10.2174/1573403X113099990030 ; PubMed Central PMCID: PMCPMC3780343. 23909638 PMC3780343

[23] Utz W, Niendorf T, Wassmuth R, Messroghli D, Dietz R, Schulz-Menger J. Contrast-dose relation in first-pass myocardial MR perfusion imaging. J Magn Reson Imaging. 2007;25(6):1131–5. 10.1002/jmri.20910 . 17520736

[24] Keijer JT, van Rossum AC, van Eenige MJ, Karreman AJ, Hofman MB, Valk J, et al Semiquantitation of regional myocardial blood flow in normal human subjects by first-pass magnetic resonance imaging. Am Heart J. 1995;130(4):893–901. . 7572601 10.1016/0002-8703(95)90092-6

[25] Manning WJ, Atkinson DJ, Grossman W, Paulin S, Edelman RR. First-pass nuclear magnetic resonance imaging studies using gadolinium-DTPA in patients with coronary artery disease. J Am Coll Cardiol. 1991;18(4):959–65. . 1894870 10.1016/0735-1097(91)90754-w

[26] Mordini FE, Haddad T, Hsu LY, Kellman P, Lowrey TB, Aletras AH, et al Diagnostic accuracy of stress perfusion CMR in comparison with quantitative coronary angiography: fully quantitative, semiquantitative, and qualitative assessment. JACC Cardiovasc Imaging. 2014;7(1):14–22. 10.1016/j.jcmg.2013.08.014 ; PubMed Central PMCID: PMCPMC4186701. 24433707 PMC4186701

[27] Kelle S, Graf K, Dreysse S, Schnackenburg B, Fleck E, Klein C. Evaluation of contrast wash-in and peak enhancement in adenosine first pass perfusion CMR in patients post bypass surgery. J Cardiovasc Magn Reson. 2010;12:28 10.1186/1532-429X-12-28 ; PubMed Central PMCID: PMCPMC2887852. 20465836 PMC2887852

[28] Limalanathan S, Eritsland J, Andersen GO, Klow NE, Abdelnoor M, Hoffmann P. Myocardial salvage is reduced in primary PCI-treated STEMI patients with microvascular obstruction, demonstrated by early and late CMR. PLoS One. 2013;8(8):e71780 10.1371/journal.pone.0071780 ; PubMed Central PMCID: PMC3747268. 23977143 PMC3747268

[29] Shetelig C, Limalanathan S, Eritsland J, Hoffmann P, Seljeflot I, Gran JM, et al Osteoprotegerin levels in ST-elevation myocardial infarction: Temporal profile and association with myocardial injury and left ventricular function. PLoS One. 2017;12(3):e0173034 10.1371/journal.pone.0173034 ; PubMed Central PMCID: PMCPMC5333871. 28253327 PMC5333871

[30] Carberry J, Carrick D, Haig C, Rauhalammi SM, Ahmed N, Mordi I, et al Remote Zone Extracellular Volume and Left Ventricular Remodeling in Survivors of ST-Elevation Myocardial Infarction. Hypertension. 2016;68(2):385–91. 10.1161/HYPERTENSIONAHA.116.07222 ; PubMed Central PMCID: PMCPMC4956675. 27354423 PMC4956675

[31] Yellon DM, Hausenloy DJ. Myocardial reperfusion injury. N Engl J Med. 2007;357(11):1121–35. 10.1056/NEJMra071667 . 17855673

[32] Wu KC. CMR of microvascular obstruction and hemorrhage in myocardial infarction. J Cardiovasc Magn Reson. 2012;14:68 10.1186/1532-429X-14-68 ; PubMed Central PMCID: PMC3514126. 23021401 PMC3514126

[33] Orn S, Manhenke C, Greve OJ, Larsen AI, Bonarjee VV, Edvardsen T, et al Microvascular obstruction is a major determinant of infarct healing and subsequent left ventricular remodelling following primary percutaneous coronary intervention. Eur Heart J. 2009;30(16):1978–85. 10.1093/eurheartj/ehp219 . 19502624

[34] Noel B, Morice MC, Garot J, Louvard Y, Tavolaro O, Dumas P, et al CMR assessment of AMI patients treated by PCI shows delays in myocardial reperfusion despite initial achievement of TIMI 3 flow. EuroIntervention. 2005;1(2):214–8. . 19758906

[35] Lombardo A, Niccoli G, Natale L, Bernardini A, Cosentino N, Bonomo L, et al Impact of microvascular obstruction and infarct size on left ventricular remodeling in reperfused myocardial infarction: a contrast-enhanced cardiac magnetic resonance imaging study. Int J Cardiovasc Imaging. 2012;28(4):835–42. 10.1007/s10554-011-9901-7 . 21643941

[36] Bodi V, Sanchis J, Lopez-Lereu MP, Nunez J, Sanz R, Palau P, et al Microvascular perfusion 1 week and 6 months after myocardial infarction by first-pass perfusion cardiovascular magnetic resonance imaging. Heart. 2006;92(12):1801–7. 10.1136/hrt.2005.077305 ; PubMed Central PMCID: PMCPMC1861306. 16803939 PMC1861306

[37] Taylor AJ, Al-Saadi N, Abdel-Aty H, Schulz-Menger J, Messroghli DR, Friedrich MG. Detection of acutely impaired microvascular reperfusion after infarct angioplasty with magnetic resonance imaging. Circulation. 2004;109(17):2080–5. 10.1161/01.CIR.0000127812.62277.50 . 15117844

[38] Nagao M, Higashino H, Matsuoka H, Kawakami H, Mochizuki T, Murase K, et al Clinical importance of microvascular obstruction on contrast-enhanced MRI in reperfused acute myocardial infarction. Circ J. 2008;72(2):200–4. . 18219154 10.1253/circj.72.200

[39] Rogers WJ Jr., Kramer CM, Geskin G, Hu YL, Theobald TM, Vido DA, et al Early contrast-enhanced MRI predicts late functional recovery after reperfused myocardial infarction. Circulation. 1999;99(6):744–50. . 9989958 10.1161/01.cir.99.6.744

[40] Lee WW, Marinelli B, van der Laan AM, Sena BF, Gorbatov R, Leuschner F, et al PET/MRI of inflammation in myocardial infarction. J Am Coll Cardiol. 2012;59(2):153–63. 10.1016/j.jacc.2011.08.066 ; PubMed Central PMCID: PMCPMC3257823. 22222080 PMC3257823

[41] Nagao M, Higashino H, Matsuoka H, Kawakami H, Mochizuki T, Uemura M, et al Analysis of microvascularity after reperfused acute myocardial infarction using the maximum slope method of contrast-enhanced magnetic resonance imaging. Radiat Med. 2008;26(5):296–304. 10.1007/s11604-008-0230-2 . 18661214

[42] Uren NG, Melin JA, De Bruyne B, Wijns W, Baudhuin T, Camici PG. Relation between myocardial blood flow and the severity of coronary-artery stenosis. N Engl J Med. 1994;330(25):1782–8. 10.1056/NEJM199406233302503 . 8190154

[43] Reinstadler SJ, Stiermaier T, Liebetrau J, Fuernau G, Eitel C, de Waha S, et al Prognostic Significance of Remote Myocardium Alterations Assessed by Quantitative Noncontrast T1 Mapping in ST-Segment Elevation Myocardial Infarction. JACC Cardiovasc Imaging. 2018;11(3):411–9. 10.1016/j.jcmg.2017.03.015 . 28624398

[44] Carrick D, Haig C, Rauhalammi S, Ahmed N, Mordi I, McEntegart M, et al Pathophysiology of LV Remodeling in Survivors of STEMI: Inflammation, Remote Myocardium, and Prognosis. JACC Cardiovasc Imaging. 2015;8(7):779–89. 10.1016/j.jcmg.2015.03.007 ; PubMed Central PMCID: PMCPMC4509710. 26093923 PMC4509710

[45] Cheng R, Wei G, Yu L, Su Z, Wei L, Bai X, et al Coronary flow reserve in the remote myocardium predicts left ventricular remodeling following acute myocardial infarction. Yonsei Med J. 2014;55(4):904–11. 10.3349/ymj.2014.55.4.904 ; PubMed Central PMCID: PMCPMC4075393. 24954317 PMC4075393

[46] de Boer RA, Pinto YM, Suurmeijer AJ, Pokharel S, Scholtens E, Humler M, et al Increased expression of cardiac angiotensin II type 1 (AT(1)) receptors decreases myocardial microvessel density after experimental myocardial infarction. Cardiovasc Res. 2003;57(2):434–42. . 12566116 10.1016/s0008-6363(02)00704-6

[47] Geshi T, Nakano A, Uzui H, Okazawa H, Yonekura Y, Ueda T, et al Relationship between impaired microvascular function in the non-infarct-related area and left-ventricular remodeling in patients with myocardial infarction. Int J Cardiol. 2008;126(3):366–73. 10.1016/j.ijcard.2007.04.042 . 17588694

[48] Bax M, de Winter RJ, Koch KT, Schotborgh CE, Tijssen JG, Piek JJ. Time course of microvascular resistance of the infarct and noninfarct coronary artery following an anterior wall acute myocardial infarction. Am J Cardiol. 2006;97(8):1131–6. 10.1016/j.amjcard.2005.11.026 . 16616013

[49] Teunissen PF, Timmer SA, Danad I, de Waard GA, van de Ven PM, Raijmakers PG, et al Coronary vasomotor function in infarcted and remote myocardium after primary percutaneous coronary intervention. Heart. 2015;101(19):1577–83. 10.1136/heartjnl-2015-307825 . 26246402

[50] Chung H, Yoon JH, Yoon YW, Park CH, Ko EJ, Kim JY, et al Different contribution of extent of myocardial injury to left ventricular systolic and diastolic function in early reperfused acute myocardial infarction. Cardiovasc Ultrasound. 2014;12:6 10.1186/1476-7120-12-6 ; PubMed Central PMCID: PMCPMC3922533. 24512272 PMC3922533

[51] Oskarsson HJ, Coppey L, Weiss RM, Li WG. Antioxidants attenuate myocyte apoptosis in the remote non-infarcted myocardium following large myocardial infarction. Cardiovasc Res. 2000;45(3):679–87. . 10728389 10.1016/s0008-6363(99)00400-9

[52] Corday E, Kaplan L, Meerbaum S, Brasch J, Costantini C, Lang TW, et al Consequences of coronary arterial occlusion on remote myocardium: effects of occlusion and reperfusion. Am J Cardiol. 1975;36(3):385–94. . 1166843 10.1016/0002-9149(75)90493-2

[53] Olivetti G, Quaini F, Sala R, Lagrasta C, Corradi D, Bonacina E, et al Acute myocardial infarction in humans is associated with activation of programmed myocyte cell death in the surviving portion of the heart. J Mol Cell Cardiol. 1996;28(9):2005–16. 10.1006/jmcc.1996.0193 . 8899559

[54] Doost Hosseiny A, Moloi S, Chandrasekhar J, Farshid A. Mortality pattern and cause of death in a long-term follow-up of patients with STEMI treated with primary PCI. Open Heart. 2016;3(1):e000405 10.1136/openhrt-2016-000405 ; PubMed Central PMCID: PMCPMC4836287. 27099764 PMC4836287

[55] Wald DS, Morris JK, Wald NJ, Chase AJ, Edwards RJ, Hughes LO, et al Randomized trial of preventive angioplasty in myocardial infarction. N Engl J Med. 2013;369(12):1115–23. 10.1056/NEJMoa1305520 . 23991625

