# Correction: MR findings of microvascular perfusion in infarcted and remote myocardium early after successful primary PCI

**DOI:** 10.1371/journal.pone.0213636

**Published:** 2019-03-06

**Authors:** The PLOS ONE Staff

There is an error in affiliation 3 for authors for Jan Eritsland and Geir Øystein Andersen. The publisher apologies for the error[s]. The correct affiliation 3 is: Department of Cardiology, Oslo University Hospital, Ullevål, Oslo, Norway.
