# Supplementary figures and images for: MR findings of microvascular perfusion in infarcted and remote myocardium early after successful primary PCI
Source: PLoS One. 2018 Nov 9;13(11):e0206723. doi: 10.1371/journal.pone.0206723 (PMC6226160; doi:10.1371/journal.pone.0206723)

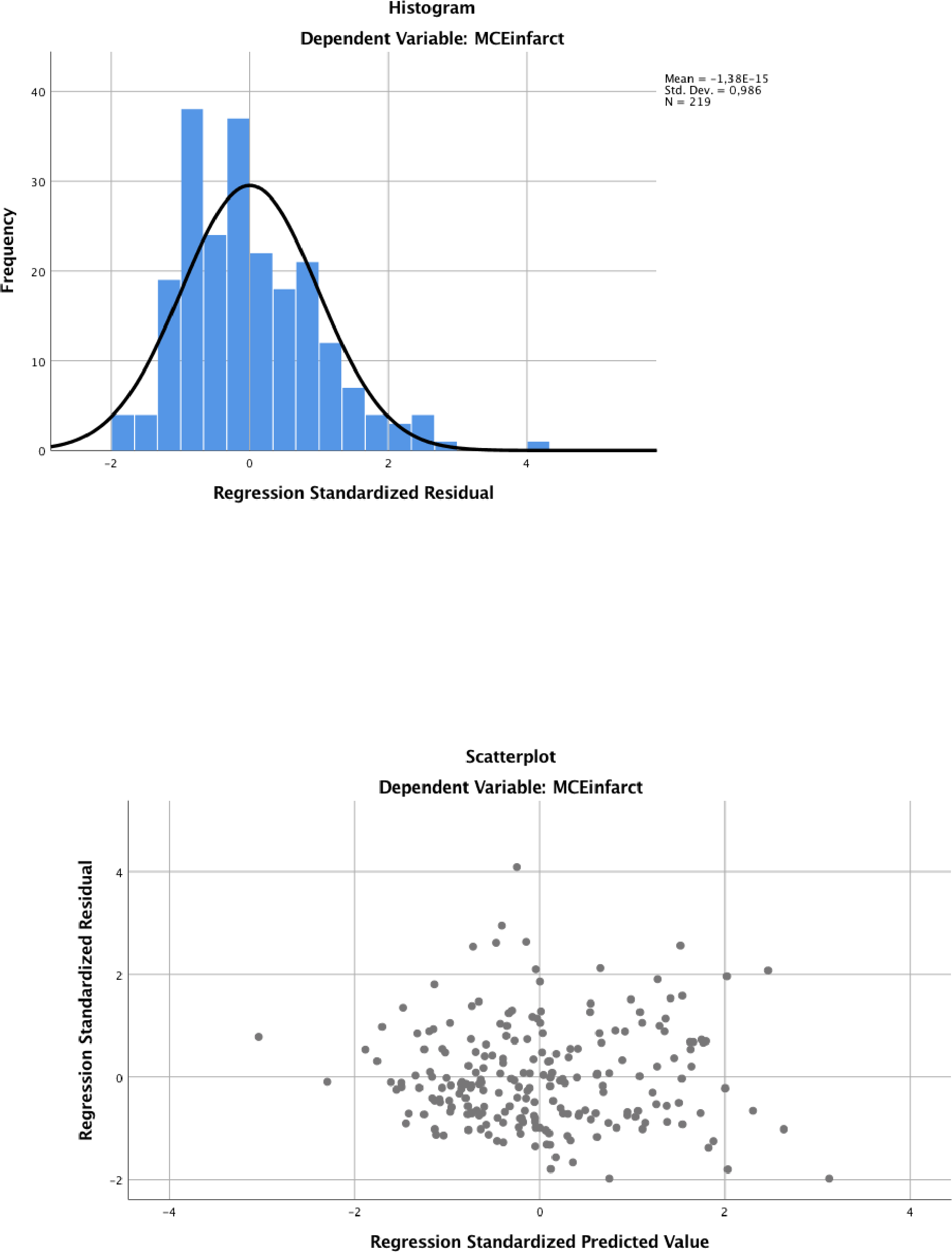

Supplement: S1 Fig — (TIF) [file pone.0206723.s001.tif]
